# Mechanisms of Rebaudioside A Degradation and Ingredient-Sweetener Interactions in Beverages during Storage

**DOI:** 10.3390/molecules27041385

**Published:** 2022-02-18

**Authors:** Benjamin S. Gelinas, Edisson Tello, Devin G. Peterson

**Affiliations:** Department of Food Science and Technology, The Ohio State University, 2015 Fyffe Rd., Columbus, OH 43210, USA; gelinas.7@osu.edu (B.S.G.); tellocamacho.1@osu.edu (E.T.)

**Keywords:** stevia, stability, sweet beverages, flavor interactions, rebaudioside A, degradation

## Abstract

The instability of rebaudioside A (Reb A) in food product applications during storage challenges their utilization. The pathways of Reb A degradation in aged acidic beverages were investigated. Three Reb A degradation compounds of known sensory importance were monitored, consisting of (**1**) a rearrangement, (**2**) a hydration, and (**3**) an epoxidation/rearrangement product. Using deuterium-labeled water (D_2_O) experiments, compounds **1**–**2** were reported to be generated by acid-catalyzed mechanisms involving the formation of a carbocation on carbon position 16, followed by either deprotonation via E_1_ elimination on C15 to form the more thermodynamically stable trisubstituted alkene (compound **1**), or by the Markovnikov addition of water via SN_1_ substitution to form a tertiary alcohol (compound **2**). Compound **3** was generated by epoxidation of the exomethylene at the C16–17 positions, followed by the opening and rearrangement of the ring to form a new alkene bond between C15–C16 and a primary alcohol on C17. Further analysis of the effect of beverage ingredients indicated the addition of caramel color significantly increased (*p* < 0.0001) the concentrations of compounds **1**–**2** compared to the aged control by 89 and 83%, respectively, whereas a specific coffee flavor and caramel color were reported to significantly reduce (*p* < 0.0001) the formation of compound **3** compared to the aged control during storage by 90 and 79%, respectively.

## 1. Introduction

Non-caloric stevia-based sweeteners, primarily based on rebaudioside A (Reb A), have increased in market utilization and consumer demand, with a projected market annual growth rate of 6.1% [1]. Part of the growth of stevia sweeteners has been driven by the decreased demand for sugar-sweetened beverages (SSB) [2,3], stemming from public concern regarding added sugar in the diet, which has been associated with an increased risk of serious health problems, including diabetes, hypertension, and heart disease [4,5]. Currently, beverages compose the largest market share of high-intensity sweeteners, including stevia, consisting of approximately one-third of the stevia market [1]. Historically, stevia has been used as a sweetener in Central and South America since before the 20th century [6]. Reb A, a diterpene glycoside isolated from the *Stevia rebaudiana* shrub native to Central and South America, is the primary sweetener used in commercial stevia-based sweeteners due to its relatively clean taste profile and high abundance in the leaves [6].

However, stevia sweeteners are not without challenges compared to sugar-based ingredients. Numerous studies have reported the chemical instability of Reb A under various conditions (light, elevated temperature, and low pH); however, characterization of the mechanisms of degradation is lacking [7,8,9]. Understanding the pathways stevia sweeteners degrade upon is of importance, as these ingredients are commonly utilized in low-pH shelf-stable products. More recently, Gelinas et al. [10], using an untargeted chemical profiling approach, identified three Reb A degradation compounds generated during storage that directly resulted in a perceivable flavor change. The three degradation compounds were identified as 13-[(2-*O*-β-d-glucopyranosyl-3-*O*-β-d-gluopyranosyl-β-d-glucopyranosyl)oxy]-*ent*-kaur-15-en-19-oic acid β-d-glucopyranosyl ester (compound **1**), 13-[(2-*O*-β-d-glucopyranosyl-3-*O*-β-d-glucopyranosyl-β-d-glucopyranosyl)oxy]-16-β-hydroxy-*ent*-kauran-19-oic acid β-d-glucopyranosyl ester (compound **2**), and 13-[(2-*O*-β-d-glucopyranosyl-3-*O*-β-d-gluopyranosyl-β-d-glucopyranosyl)oxy]-17-hydroxy-*ent*-kaur-15-en-19-oic acid β-d-glucopyranosyl ester (compound **3**). Others have reported that the degradation of Reb A can reach levels that result in a perceivable loss of sweetness intensity [8].

In the current study, the pathways resulting in the formation of Reb A degradation compounds **1**–**3** were investigated in aqueous beverage samples during storage using isotope labeling (deuterium oxide) and computational molecular energy calculations. In addition, the impact of common beverage ingredients utilizing Reb A, flavoring materials, and color on the formation of compounds **1**–**3** was examined.

## 2. Results and Discussion

### 2.1. Mechanism of Reb A Degradation and Molecular Energy Calculations

**Figure 1:**
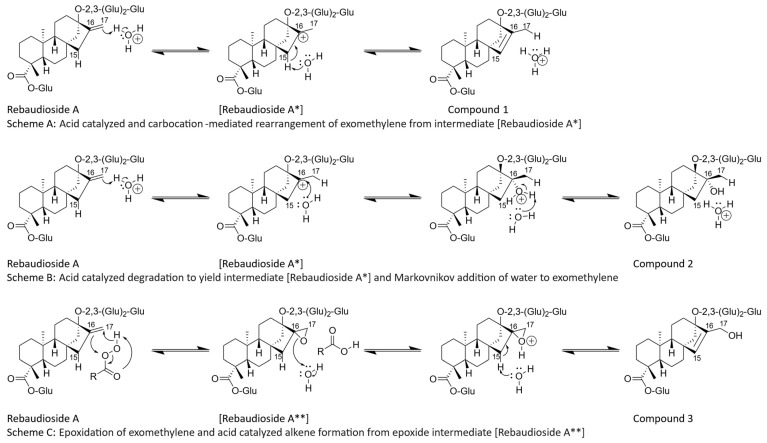
Mechanisms of rebaudioside A degradation.

**Figure 2:**
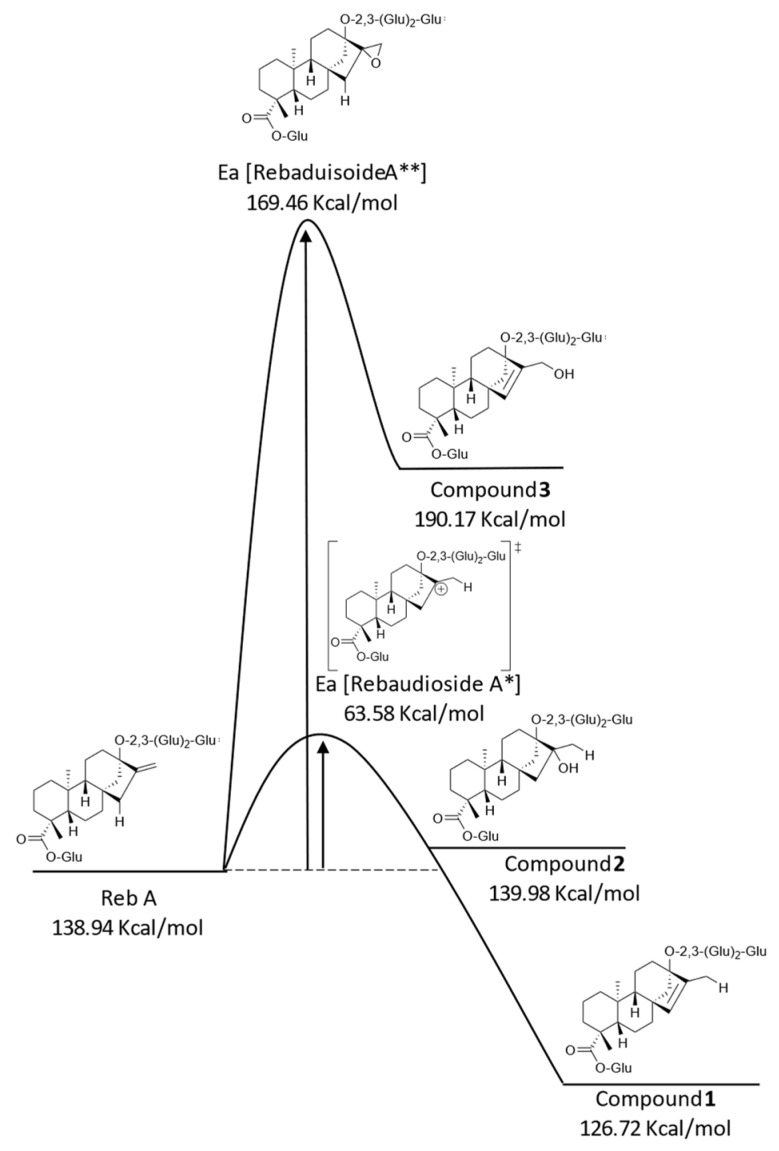
Potential energy diagram of Reb A and degradation compounds **1**–**3**.

The degradation of Reb A has been reported to be catalyzed by lower pH conditions [8]. Based on this observation, it was hypothesized that the exomethylene group at C16–C17 on Reb A was labile to acid degradation (Figure 1, Scheme A). A likely acid-catalyzed thermodynamic rearrangement involves the carbocation intermediate, resulting in the formation of compound **1**. This proposed pathway is in agreement with previous studies that identified the major degradation product of Reb A under acidic conditions as compound **1** [7,8]. To form the carbocation intermediate, the olefin of Reb A must react with a proton from the aqueous environment, leading to the formation of a relatively stable, tertiary carbocation (Rebaudioside A*) (Figure 1, Scheme A) requiring 63.58 kcal/mol of activation energy (Figure 2), and the acidic proton on C15 is removed by a hydrogen transfer to water via an E_1_ elimination mechanism, yielding compound **1**.

**Figure 3:**
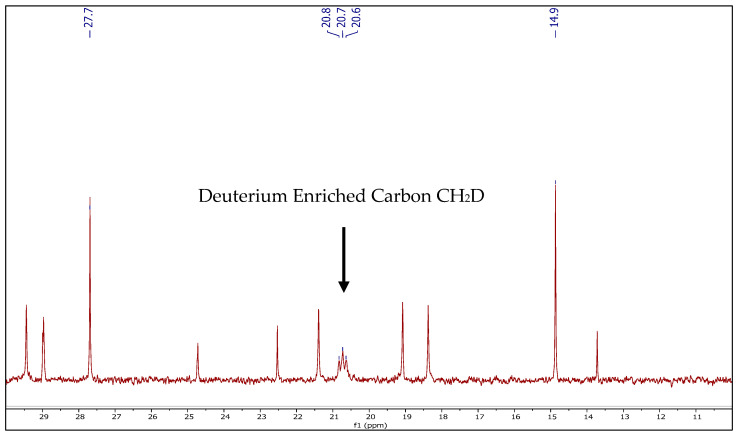
^13^C NMR analysis of deuterated rebaudioside A degradation compound **2d** highlighting triplet on C17.

Compound **1**, compared to Reb A, was more thermodynamically favored with an energy 12.22 kcal/mol lower, indicating this reaction would be expected to continue over time. To confirm the proposed pathway of compound **1** formation (Figure 1, Scheme A), an isotopic-labeling study was conducted with Reb A aged in deuterium-labeled water (D_2_O). NMR analysis of the compound **1** formed during storage in D_2_O revealed a loss of 1H on the C17 methyl located at 1.56 ppm (CH_2_-d) in the proton spectrum (Table 1), along with the presence of a new triplet signal resonating at 12.4 ppm (Figure 3) in the ^13^C spectrum, consistent with the proposed mechanism (Figure 1, Scheme A). Therefore, the generation of compound 1 in D_2_O incorporated a single deuterium atom on the C17 methyl group to form compound **1d** (Figure 2). Furthermore, an LC–MS analysis of compound **1d** generated in D_2_O similarly revealed the addition of one deuterium atom in the elemental composition (*m*/*z* 966.4271 [M − H]^−^, C_44_H_69_O_23_D).

The proposed formation of compound **2** (Figure 1, Scheme B) was closely related to the generation of compound **1**, which is formed via SN_1_ substitution at the same carbocation intermediate (Rebaudioside A*) and calculated at 63.58 kcal/mol of activation energy (Figure 2). The Markovnikov addition of water to the carbocation intermediate (Rebaudioside A*) would lead to the formation of compound **2**, a product with an energy 1.04 kcal/mol higher than Reb A, indicating the formation of compound 2 would be less favorable with respect to that of compound **1** (Figure 2). This pathway was in agreement with the NMR and accurate-mass MS analysis of compound **2d** (Figure 2) isolated from the aged samples with D_2_O. The formed tertiary hydroxyl (-OD) on C16 on compound **2** (Figure 1, Scheme B) would rapidly exchange with hydrogen from the aqueous solvents during the prep-LC–MS isolation step; however, the C-D on C17 was detected by NMR and MS analysis. A review of the proton spectrum of compound **2** for C17 revealed the loss of 1H at 1.31 ppm (Table 1), and the ^13^C spectrum indicated a triplet at 21.0 ppm (Figure 3), confirming the proposed mechanism (Figure 1, Scheme B). An LC–MS analysis of compound **2d** (Figure 2) indicated the addition of one deuterium atom in the elemental composition (*m*/*z* 984.4411 [M − H]^−^, C_44_H_71_O_24_D).

The final Reb A degradation product, compound **3** (Figure 1, Scheme C), was proposed to be formed by an oxidative pathway, in contrast to the acid-catalyzed formation of compounds **1**–**2**. For compound **3** to form via a carbocation intermediate product, a 1,3-hydride shift or hydride loss was needed. As 1,3-hydride shifts are spin-forbidden, thus requiring two consecutive 1,2-hydrides shifts to form compound **3**, this mechanism seemed unlikely [11]. Furthermore, compound **3** would require the anti-Markovnikov addition of hydroxyl to form a primary alcohol on C17, which seemed unlikely given the mildly acidic and temperature conditions (35 °C). Thus, the compound **3** mechanism was suggested as a two-step reaction: first, the epoxidation of the exomethylene at the C16–17 positions requiring 169.46 kcal/mol of activation energy (Figure 2), followed by the E1 elimination of the acidic proton on C15 (Rebaudioside A**), and a subsequent and concerted ring opening to form a new alkene bond between C15–C16 and a primary alcohol on C17 (Figure 1, Scheme C), with an overall energy of 190.17 kcal/mol, 51.23 kcal/mol higher than the starting material, Reb A (Figure 2). NMR and mass spectra analysis of compound **3** generated from Reb A and aged in D_2_O were consistent with the epoxidation mechanism, as no additional deuterium atoms were detected in MS, ^1^H NMR, or ^13^C NMR. No deuterium atoms were expected to be incorporated into compound **3** except for the hydroxyl at C17, which would be exchanged during isolation by prep-LC–MS. Epoxide intermediates under acidic, aqueous conditions are known to form trans diols, which were not observed experimentally. Molecular steric effects may have prevented the formation of a diol, or the diol may be the less thermodynamically favored product.

**Figure 4:**
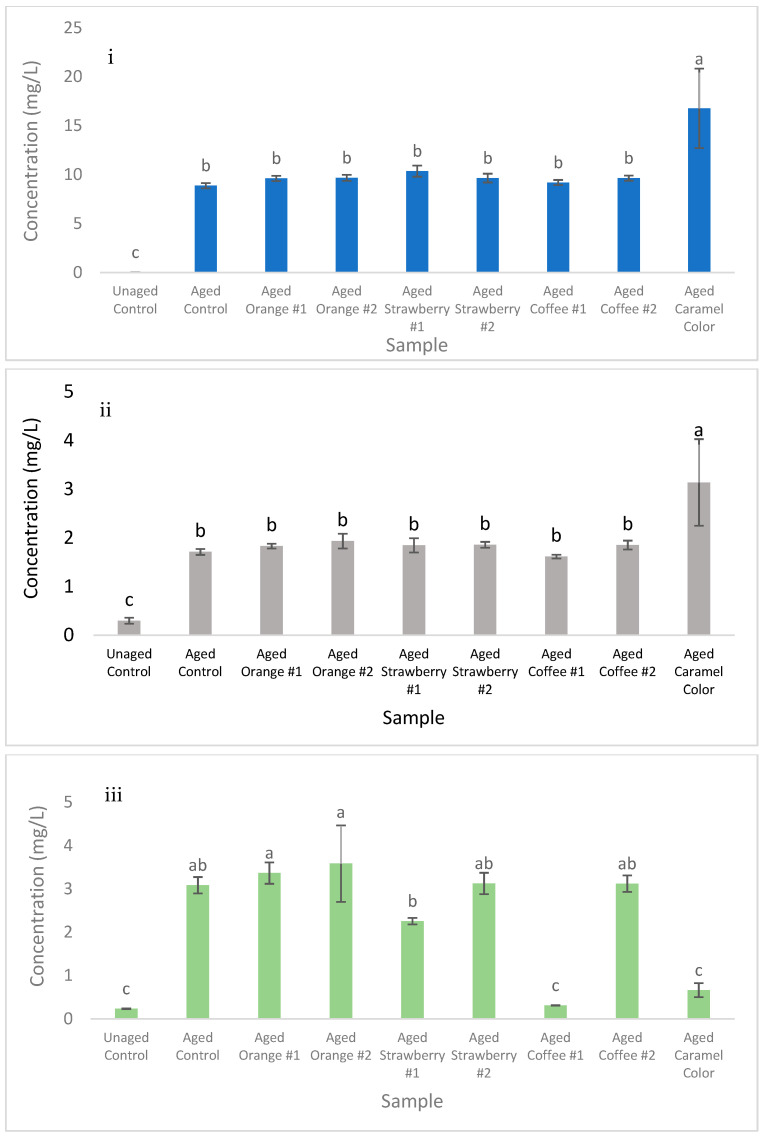
Quantification of rebaudioside A degradation compounds **1** (**i**), **2** (**ii**), and **3** (**iii**) in unaged and aged (6 weeks, 35 °C) acidified aqueous samples consisting of rebaudioside A (control samples) or rebaudioside A with different flavors (two orange, two strawberry, two coffee) and color (caramel). Different letters indicate significant differences at α = 0.05 Tukey HSD.

Therefore, two major pathways were reported for the formation of Reb A degradation compounds **1**–**3** that involved either an acid-catalyzed carbocation re-engagement, or an epoxidation of the exomethylene at C16–C17 with a subsequent ring-opening promoted by E_1_ elimination (Figure 1). This implicated the exomethylene moiety at the C16–17 position on Reb A as a key chemical functional group that impacts flavor stability. The quantities of the degradation compounds **1**, **2**, and **3**, as generated in acidic aqueous samples stored for six weeks at 35 °C, were 8.9, 1.4, and 2.9 mg/L, respectively (Figure 4i–iii, control samples). The yields generated for compounds **1**–**3** are likely driven by competing mechanisms, those being the activation and potential energy of each product. Interestingly, given that compounds **1** and **2** form via the same intermediate carbocation product (Figure 2), the formation of compound **1** was strongly favored at approximately 6.3 times the rate of compound **2** in the control samples. This difference in formation was in agreement with the calculated lower potential energy of compound **1** compared to compound **2**.

### 2.2. Beverage Ingredient–Rebaudioside A Interactions

Based on the two major pathways of instability reported for Reb A, involving an acid-catalyzed carbocation re-engagement or epoxidation/rearrangement reaction, it was hypothesized that ingredients common in beverages, such as flavorings or coloring agents, could potentially impact the formation of compounds **1**–**3**. Caramel color Type IV (E150d) is commonly used in soft drinks [12,13]. Flavoring materials typically contain a wide variety of chemical compounds of different classes that could alter the stability of Reb A. Based on the mechanisms in Figure 1, Schemes A–C, we hypothesized that flavoring or color compounds with an antioxidant function could scavenge the peroxide intermediates needed to form compound **3**, and that compounds with a Lewis acid and base function could stabilize the carbocation intermediate (Rebaudioside A*) (Figure 3). Similarly, caramel color has been characterized as having acidic function groups that could catalyze the generation of compounds **1**–**2** [14].

The concentrations of Reb A degradation compounds **1**–**3** in nine acidified aqueous samples consisting of Reb A with flavoring materials, including two strawberry, two orange, two commercial coffee flavors, and one caramel color (E150d), and two control samples (Reb A only, unaged and aged), are shown in Figure 4. Only the addition of caramel color significantly (*p* < 0.0001) increased the concentration of compounds **1**–**2** compared to the aged control samples by 89 and 83%, respectively (Figure 4i and ii)**.** However, for compound **3**, one coffee flavor and the caramel color were reported to significantly (*p* < 0.0001) reduce the formation of compound **3** during storage compared to the aged control by 80 and 90%, respectively (Figure 4iii). Furthermore, no significant differences were observed between the unaged control and the caramel color and coffee flavor #1 (*p* = 0.80 and *p* = 0.92), indicating the complete suppression of compound **3** formation during storage in samples containing these ingredients.

The addition of caramel color was reported to catalyze the degradation of Reb A under an equivalent pH value as the control sample. Caramel colors are known to contain Lewis acids such as furans and caramelans [14,15,16], which can promote the proton addition on C-17 and the subsequent formation of Rebaudioside A* intermediate, a tertiary carbocation on C16 (Figure 1, Schemes A and B). Whereas Lewis bases could either deprotonate C15 leading to the formation of compound **1** (Figure 1, Scheme A) or stabilize the tertiary carbocation on C16 of the Rebaudioside A* intermediate, allowing the formation of compound **2** (Figure 1, Scheme B). Both mechanisms would be anticipated to catalyze the formation of degradation compounds **1**–**2**.

The noted inhibition of compound **3** generation during storage by the caramel color and a specific coffee flavor compared to the control sample (Figure 4iii) indicated these materials suppressed epoxidation of Reb A. Interestingly, the amounts of compound **3** in these aged samples were not significantly different from the unaged control. Non-enzymatic browning reaction products in the caramel color and coffee materials likely functioned as antioxidants, scavenging the peroxide intermediates needed for the epoxidation of Reb A to form (Rebaudioside A**), and stabilizing rebaudioside A during storage [17]. Polyphenolic compounds commonly present in coffee products are well documented for scavenging peroxide intermediates and preventing oxidation in foods [18]. Caramel colors are known to contain a variety of melanoidins and caramelans that exhibit antioxidant activity [19], which was suggested to decrease the formation of compound **3** during storage via peroxide scavenging in the current study.

## 3. Materials and Methods

### 3.1. Preparation of Deuterium-Enriched Rebaudioside A Degradation Products

A mixture of 300 g deuterium oxide (99% D) (Sigma Aldrich, Saint Louis, MO, USA), 540 mg rebaudioside A, 135 mg citric acid, 27 mg malic acid, 54 mg potassium sorbate, and 27 mg sodium benzoate (Fisher Scientific, Waltham, MA, USA) was prepared in a 250 mL bottle (pH = 3.5). Dry nitrogen was purged to remove oxygen, and the sample was stored at 35 °C for 6 weeks. Samples were prepared aseptically in a climate-controlled hood and monitored for microbial growth during storage.

### 3.2. Solid-Phase Extraction of Rebaudioside A Degradation Products

Aged sample (150 mL) was loaded onto each preconditioned HLB SPE cartridge (6 g, 35 cc, Waters Co., Milford, MA, USA), followed by a 60 mL wash with 5% methanol in water, followed by a 60 mL elution (5% methanol in acetone). The organic elution solvents were evaporated under vacuum, reconstituted in 25% acetone in water, and injected onto the preparative chromatography system as described below.

### 3.3. First-Dimension Preparative Liquid Chromatography

A 2767 auto-purification system with 2525 Binary Gradient Manager (Waters Co.) coupled to a Micromass (Waters Co., Milford, MA, USA) triple quadrupole detector (QQQ) was used for compound purification. The system was equipped with an OBD C-18 prep column (50 × 50 mm, 5 µm, Waters Co., Milford, MA, USA.) and operated at a flow rate of 100 mL/min. The injection volume was 6.0 mL. Acidified water (0.1% formic acid, *v*/*v*) and acidified acetone (0.1% formic acid, *v*/*v*) were used as mobile phases A and B, respectively. The gradient was programmed as follows: 0 min, 25% B; 1 min, 35% B; 6 min, 55% B; 7 min, 95% B; 9 min, 25% B. Initial conditions were held for 2 min as a re-equilibration step. The mass spectrometer was operated in single ion monitoring (SIM) mode for the [M − H]^−^ masses of interest: 965.4 (compound **1**), 983.4 (compound **2**), and 981.3 (compound **3**). Mass spectrometer was operated in negative ion mode with the following source settings: capillary voltage 2.5 kV, cone voltage 30 V, source temperature 150 °C, desolvation gas temperature 350 °C, cone gas flow rate 60 L/h, and desolvation flow rate 500 L/h. The fraction collector was set to time-based collection using the retention time window of the masses of interest. Solvent was removed in vacuo using rotary evaporators or Genevac rocket synergy (SP scientific, Warminster, UK), followed by lyophilization (SP Scientific, Warminster, UK) to dried powders.

### 3.4. Second-Dimension Isolation of Deuterium-labeled Compounds ***1**–**3***

A Waters 2795 Alliance (Waters Co., Milford, MA, USA) high-performance liquid chromatography (HPLC) system, consisting of a quaternary pump, autosampler, and column heater coupled to a Micromass triple quadrupole detector (Waters Co., Milford, MA, USA), was used for the separations. The flow rate was 4.0 mL/min. Injection volume ranged from 250 to 350 µL, and the column temperature was 30 °C. Mass spectrometer operated in negative ion mode with the following source parameters: capillary voltage 3.0 kV, cone voltage 30 V, source temperature 120 °C, desolvation gas temperature 350 °C, cone gas flow rate 60 L/h, and desolvation flow rate 600 L/h. A Waters Fraction Collector III was set to mass-based collection for ions of interest (single ion monitoring mode) triggered at the ion intensity of 500,000 counts or more. All second-dimension separations were carried out on a Waters Atlantis T3 (10 × 250 mm, 5 μm) semi-preparative column. Acidified water (0.1% formic acid, *v*/*v*) and acidified acetone (0.1% formic acid, *v*/*v*) were used as mobile phases A and B, respectively.

For compound **1**, the custom solvent gradient program was as follows: 0 min, 45% B; 12 min, 49% B; 12.01 min, 95% B; 15 min, 95% B; 15.01 min, 45% B. For compound **2**, the custom solvent gradient program was as follows: 0 min, 40% B; 15 min, 46.3% B; 15.01 min, 95% B, 18 min, 95% B; 18.01 min 40% B. For compound **3**, the custom solvent gradient program was as follows: 0 min, 35% B held isocratic for 8 min; 8.01 min, 95% B; 11 min 95% B; 11.01 min, 35% B. A three-minute re-equilibration step was carried out at initial conditions between injections. Final isolates were pooled, and the solvent was removed in vacuo using rotary evaporators, followed by lyophilization (SP Scientific, Warminster, UK) to dried powders.

### 3.5. Nuclear Magnetic Resonance Spectroscopy

A Bruker Advance III Ascent 700 MHz NMR with SampleJet and 5 mm Triple Resonance Observe Probe was used for NMR analysis (Bruker, Billerica, MA, USA). ^1^H and ^13^C spectra were collected for Reb A, compounds **1**–**3**, and also their deuterated isotopes. All compounds were taken in DMSO-*d*6.

### 3.6. Rebaudioside A Beverage Systems

A total of eight beverage sample sets were prepared, composed of two strawberry flavors, two orange flavors, two coffee flavors, one caramel color (E150D), and a no-flavor-added control. The stevia extract (98% Reb A) was incorporated at 200 mg/L. Beverage components included flavor (1000 mg/L), citric acid (500 mg/L), malic acid (100 mg/L), potassium sorbate (200 mg/L), and sodium benzoate (100 mg/L). Three replicates were prepared for all samples.

Samples were sterilized using a 0.22 μm filter prior to sample aging to rule out microbial growth as a source of flavor change and to assure the beverages were suitable for human consumption. Clear glass bottles (500 mL) were pre-sterilized in an autoclave (121 °C for 20 min) before transferring into a sterile glove box that had been cleaned and sterilized using ethanol and UV light. A peristaltic pump using Masterflex Pharma Pure (Cole Parmer, Vernon Hills, IL, USA) tubing was used to pass the sample through a pre-sterilized 0.22 μm filter (Duarpore, Millipak 20) that was purchased from EDM Millipore (Billerica, MA, USA). Samples were then subjected to accelerated storage at 35 °C for six weeks (aged) or stored at −35 °C (non-aged) control. Aged samples were monitored for aerobic total count, coliform, and yeast/mold using Petrifilm (3M, Saint Paul, MN, USA) plates incubated at 37 °C for 48 hrs for aerobic plate count, and coliform or ambient temperature for yeast/mold. No colonies were detected on any aged sample for any media following the incubation period.

### 3.7. Quantification of Rebaudioside A Degradation Products

A stock solution containing 64 mg/L of compounds **1**–**3** was prepared in a triplicate. An 8-point serial dilution was performed using 500 µL stock and 500 µL water. A Waters Cortecs C18+ (2.1 × 100 mm, 1.6 µm) column with H-class Acquity UPLC system containing quaternary solvent manager (QSM), sampler manager (FTN), and column manager coupled to a Waters TQS mass spectrometer was used for quantification. Acidified water (0.1% formic acid, *v*/*v*) and acidified acetonitrile (0.1% formic acid, *v*/*v*) were used as mobile phases A and B, respectively. The gradient was programmed as follows: 0 min, 25% B; 1 min, 35% B; 6 min, 55% B; 7 min, 95% B; 9 min 95% B; 9.01 min, 25% B. Initial conditions were held for two minutes as an equilibration step. The column temperature was 40 °C, the column flow rate was 0.5 mL/min, and the injection volume was 2 µL. Data were collected using multiple reaction monitoring (MRM) mode using the following optimized collision energies and transitions: compound **1** (965.4 → 803.40, CE: 34v), compound **2** (983.47 → 821.57, CE: 25V), and compound **3** (981.39 → 819.39, CE 36V). The mass spectrometer was operated in negative ion mode with the following source parameters: capillary voltage 2.5 kV, cone voltage 20 V, source temperature 150 °C, desolvation gas temperature 500 °C, cone gas flow rate 150 L/h, and desolvation flow rate 1000 L/h.

### 3.8. Molecular Energy Calculations

Potential energy minimizations were carried out using ChemDraw Professional 3D Version 19.1 with a minimum RMS gradient of 0.01 and a maximum number of 10,000 iterations.

## 4. Conclusions

The two main mechanisms of Reb A degradation in acidic aqueous samples during storage were identified as (1) an acid-catalyzed carbocation intermediate, forming compound **1** via the E_1_-elimination rearrangement of the alkene, or the SN_1_ Markovnikov addition of water to form compound **2**, and (2) an epoxidation with a subsequent ring opening to form compound **3**. Furthermore, common beverage ingredient interactions were demonstrated to impact the stability of Reb A. The caramel color significantly increased the concentrations of compounds **1**–**2**, whereas a specific coffee flavor and caramel color were reported to reduce the formation of compound **3**. Further research is needed to understand the structure–function activity of the noted ingredient interactions on the stability of Reb A. An improved understanding of the mechanisms of Reb A instabilty is needed to optimize the flavor performance.

## Figures and Tables

**Table 1:** ^1^H and ^13^C NMR chemical shifts in deuterated rebaudioside A degradation compounds **1** and **2**.

Carbon Position	Compound 1d		Compound 2d	
	δ_C_, mult	δ_H_, (*J* in Hz)	δ_C_, mult	δ_H_, (*J* in Hz)
17	12.1, CH_2_D, t	1.56, s, 2H	20.7, CH_2_D, t	1.31, s, 2H
18	28.1, CH_3_	1.07, s, 3Hz	27.7, CH_3_	1.25, s, 3H
20	0.81, s, 3H	14.9, CH_3_	14.9, CH_3_	0.91, s, 3H

## Data Availability

Not applicable.

## Sample Availability

Samples of the compounds are not available from the authors.
